# Expressing emotions through music

**DOI:** 10.1098/rstb.2025.0131

**Published:** 2026-09-24

**Authors:** Vesa Putkinen, Maya Rassouli, Lauri Nummenmaa

**Affiliations:** Turku PET Centre, Turku, Southwest Finland, Finland

**Keywords:** emotion, music, brain, communication, synchrony

## Abstract

Music evokes strong emotions across listeners although it conveys no biologically relevant information regarding the survival challenges in the environment. Here we review the neurobiology and psychology of emotional communication through music, focussing on the functions and mechanisms of musical emotional expressions in social interaction. We also extend our analysis of the communicative space beyond the pure acoustic features to the interaction of musical emotional cues with other verbal and non-verbal communication channels. Activation likelihood estimation meta-analysis of neuroimaging studies shows functional overlap for brain systems involved in music production and emotions. Musical emotion communication, however, differs from other common expressive channels (faces and bodies, utterances and semantics) in its timescale, context, spontaneity and interactivity. Musical emotional communication can be modulated by affective information conveyed through verbal and other non-verbal channels. This makes music a flexible and culturally malleable means of context-dependent emotional communication that is particularly well suited for facilitating large-scale social affiliation and bonding.

This article is part of the theme issue ‘Mechanisms, development, phylogeny and functions of emotional expressions’.

## 1. Introduction

Music is culturally universal, has existed since prehistory and is specific to humans [1]. People engage in music for various purposes such as fostering interpersonal relationships and building and expressing personal identity [2–4]. The majority of these functions, however, share a common component—emotionality. Nearly 90% of adults listen to music every day, and practically everyone (98%) states that music supports their mental and physical health by providing emotion-related benefits such as stress relief or relaxation [5]. Music also serves as a medium for the performers to express and communicate emotions. In one survey where subjects were required to select from a comprehensive list of options what they thought music in general expresses, every single participant chose ‘emotions’ as an expression of musicality [6]. Performers also consider expressivity in music as a tool for increasing the affective impact of the music, and a majority (83%) of musicians report that they deliberately try to express specific emotions during their performances [7].

Emotions act as survival intelligence, organizing human and animal behaviour by adjusting their actions across multiple physiological and behavioural scales [8,9]. These physiological and psychological states may also be communicated to others through various emotional expressions to convey behavioural intentions and increase the predictability of social interaction [10]. Facial and bodily expressions of emotions have probably evolved to facilitate rapid social communication and negotiation in situations that acutely affect individuals' safety, survival or reproduction success [10]. The capacity to infer emotional qualities from music is convergent across cultures, as basic emotions can be recognized without prior familiarity with the musical culture [11]. Yet, whereas facial expressions and emotional vocalizations may reflect the individual's internal states that enable perceivers to adjust their behaviour accordingly, music often expresses emotion in a more symbolic and stylized form. Musical expression also allows the induction and sharing of emotions across a large number of listeners and performers through empathy and emotional contagion. Music-induced emotions are typically detached from immediate real-life events, and emotions are rarely expressed through music in survival salient situations. Therefore, it is likely that emotional communication through music also serves different adaptive functions than, for example, emotional expression with faces or bodily postures.

As music is unique to humans [1], it is likely that musical emotional expressions support functions that are unique or at least uniquely important for humans. Recent evolutionary accounts of music have focused on its social functions such as promoting bonding or credibly signalling parental care and coalition strength [12,13]. While human capacity for recognizing emotions from music has been extensively studied [6,14], the psychological and neural mechanisms involved in generating the musical emotional communication have remained poorly specified. Here, we review the neurobiology and psychology of emotional communication through music.

Emotional communication refers to the social function of expressive behaviour in coordinating, signalling or regulating affective states within interpersonal contexts. Emotional expression in turn refers to the performer's musical behaviour, including systematic modulations of sound and movement which produce structured expressive cues empirically associated with particular emotion judgments in a probabilistic rather than one-to-one manner. Emotional communication does not, however, imply a perfect correspondence between the performer's intended emotion and the emotion perceived by listeners, as expressive cues may be interpreted differently depending on context, prior experience or cultural background. We focus on the functions and mechanisms of musical emotional expression in social interaction and extend our analysis beyond purely acoustic features to the interaction of musical cues with other verbal and non-verbal communication channels. At the same time, listeners' agreement in emotional interpretation provides an important constraint on which affective qualities can be reliably conveyed through music. Accordingly, we draw on studies of listener agreement when they inform our discussion of emotional expression in music.

## 2. Music as emotional communication

Communication of emotions through music is accurate. Meta-analyses have shown that basic emotions (happiness, anger, sadness, fear, tenderness) can be expressed through music as accurately as through facial or vocal expressions, with raw accuracy scores exceeding 0.7 [15]. Meta-analyses also show that music consistently engages the brain's affective circuits [16,17] and pattern recognition studies show that subjective emotions evoked by music can also be decoded accurately from haemodynamic signals, supporting the discrete nature of musical emotional experiences [18]. However, decoding accuracy is highest in auditory and motor cortices, indicating that auditory–motor representations encode structured, emotion-category-specific information associated with music-evoked affect, whereas such information was not reliably decodable from canonical limbic emotion circuits (see also [19]). In line with this, different music-evoked emotions elicit culturally consistent bodily feeling fingerprints that closely match to those evoked by survival-related events [9,20] which may reflect the engagement of autonomic systems and preparation of the body for action. Research on musical emotions has typically focused either on a few discrete categories such as happiness, sadness or fear, or on dimensions, typically valence and arousal. While this approach has practical advantages, it is widely acknowledged that music can communicate and elicit emotions that extend beyond a few basic categories, including aesthetic emotions such as awe, nostalgia or being moved [14]. Consequently, music-specific models have been proposed to account for these musical experiences [21–23].

Perception-oriented accounts have established specific acoustic features that predict emotion ratings by passive listeners. Among these, the influential ‘common code’ hypothesis [6] proposes that music and vocal expression of emotion rely on shared acoustic cues to convey emotion. Cross-cultural studies indicate that some aspects of emotion perception in music may be largely culturally invariant while others are substantially influenced by cultural learning: basic emotions such as happiness, sadness and fear are recognized above chance across cultures, whereas more complex states (e.g. peace, longing, spirituality) are less reliably perceived [11,24,25]. Recognition is also generally more accurate for culturally familiar music, reflecting an in-group advantage. Acoustic analyses further suggest that the cues performers use and listeners rely on, overlap substantially with those known from emotional speech, supporting the view of a shared ‘common code’ across modalities.

Musicians vary timing, amplitude and timbre of their playing as these deviations are critical for delivering the aesthetic emotional experience, similarly to how prosody in speech is used for altering the interpretation of the semantic meaning [26]. Experimental studies have shown that these characteristics and particularly timing and amplitude, influence the emotions communicated. However, this effect is strongly dependent on the listeners' musical training, pointing towards a learning-based mechanism in interpreting the expressivity conveyed by the performer [27] and particularly the emotional connotations of higher-level musical features such as the major–minor distinction, with no equivalent in speech appearing to arise through enculturation [28,29]. Taken together, these findings suggest that universality in musical emotion perception is the strongest for basic emotions and tied to relatively low-level cues such as tempo and roughness [30], whereas the perception of more complex emotions may depend more strongly on cultural learning. Yet, more recent large-scale work showed that both United States and Chinese listeners consistently distinguished at least 13 categories of musically induced feelings, including triumph and erotic desire, suggesting that cross-cultural commonalities can extend beyond basic emotions [14], challenging the older data indicating that cross-cultural agreement is the strongest for basic emotion categories and decreases for more fine-grained distinctions [25].

## 3. What makes music a special emotional signal

Emotional expression through music shares some similarities with the other verbal and non-verbal emotion communication channels (table 1) yet exhibits distinctive characteristics, although these should be understood as broad tendencies rather than universal properties of all musical practices. First, facial and bodily expressions operate on sub-second temporal scales [31], whereas music is more similar in timescale to spoken and written language (though not in semantic or propositional structure), with affective cues unfolding in timescales ranging from seconds to minutes. Although emotions can be accurately recognized from sub-second musical snippets [32], the inherent communicative time window of musical pieces is significantly longer (tens of seconds to minutes) and short musical bursts are rarely used for communicative purposes. Emotional expression in music is partly based on predictive processes and rhythmic entrainment that emerge only over time with the unfolding of musical structure [33,34]. Unlike other expressive channels, music can sustain and deepen affective engagement through repetition—a process that by definition takes time: whereas we rarely repeat the same facial expression or utterance over and over, a musical phrase may recur many times within a piece, allowing emotion to unfold gradually.

**Table 1.** Basic characteristics of musical emotional expressions in relation to other primary ways of expressing emotions. (Note that musical performance can contain affective elements from all the other communication channels as well. The distinctions shown here thus reflect prototypical tendencies rather than absolute categories.)

modality	timescale	context of use	spontaneity	interactivity
**facial and bodily expressions**	sub-second	dyads and small groups	spontaneous and planned	bidirectional
**utterances**	sub-second	dyads and small groups	spontaneous and planned	bidirectional
**semantics**	seconds to minutes	dyads, small and large groups	spontaneous and planned	bidirectional
**music**	seconds to minutes	small and large groups	planned	unidirectional or parallel

Second, facial and bodily expressions as well as verbal and semantic emotional communications are typically used in dyads or small groups, whereas musical communication readily scales to large groups and is often directed at larger audiences. ^1^ One notable exception is, however, the ubiquitous practice of infant-directed singing such as lullabies which are typically confined to dyadic contexts and serve to calm and strengthen bonds between the parent and child [12].

Third, whereas all other modalities for emotional expression range from purely spontaneous and automatic to carefully planned displays, musical expression occupies the deliberate end of the spectrum. In adults, musical expression is rarely spontaneous and instead requires deliberate initiation and sustained motor organization, involving voluntary retrieval of previously learned material. By contrast, facial expressions occur routinely in everyday contexts [35], and smiling or laughing can be triggered spontaneously in ways that musical behaviour rarely is. One study found that spontaneous singing occurred only rarely and only in one-fourth of study participants and these constituted only 4% of the recorded sound episodes [36]; these estimates match closely with retrospective surveys [5]. Naturalistic recordings from toddlers, however, indicate that young children routinely and spontaneously engage in singing both improvised and learnt songs, particularly during free play. Interestingly, spontaneous singing alone was far more common than social singing with others, and self-singing was also more improvisatory rather than the ‘performative’ social singing that consists mainly of learned songs [37]. This indicates that already young children understand the communicative and social value of music, and that they begin to shape their music-making to match the social communicative norms.

Fourth, whereas emotional communication through other verbal and non-verbal channels is based on interactive sensorimotor loops where senders and receivers constantly update their expressions based on the messages they encode from others [38], musical communication is typically unidirectional or parallel. Musicians playing in an ensemble adjust tonality, pitch and rhythm with each other, but repeated exchange of emotional musical signals that would iteratively influence the emotional nature of the musical signals is relatively uncommon. Although examples of such bidirectional exchange exist—such as expert jazz musicians engaging in mutual adaptation to each other's improvised phrases, rap battles or call-and-response traditions found in many non-Western musical cultures—even in these cases, musical turn-taking typically unfolds over longer timescales and within relatively fixed stylistic and structural constraints, lacking the rapid, open-ended, turn-by-turn bidirectionality that characterizes spoken language.

## 4. Brain mechanisms for music production and emotions

**Figure 1.**
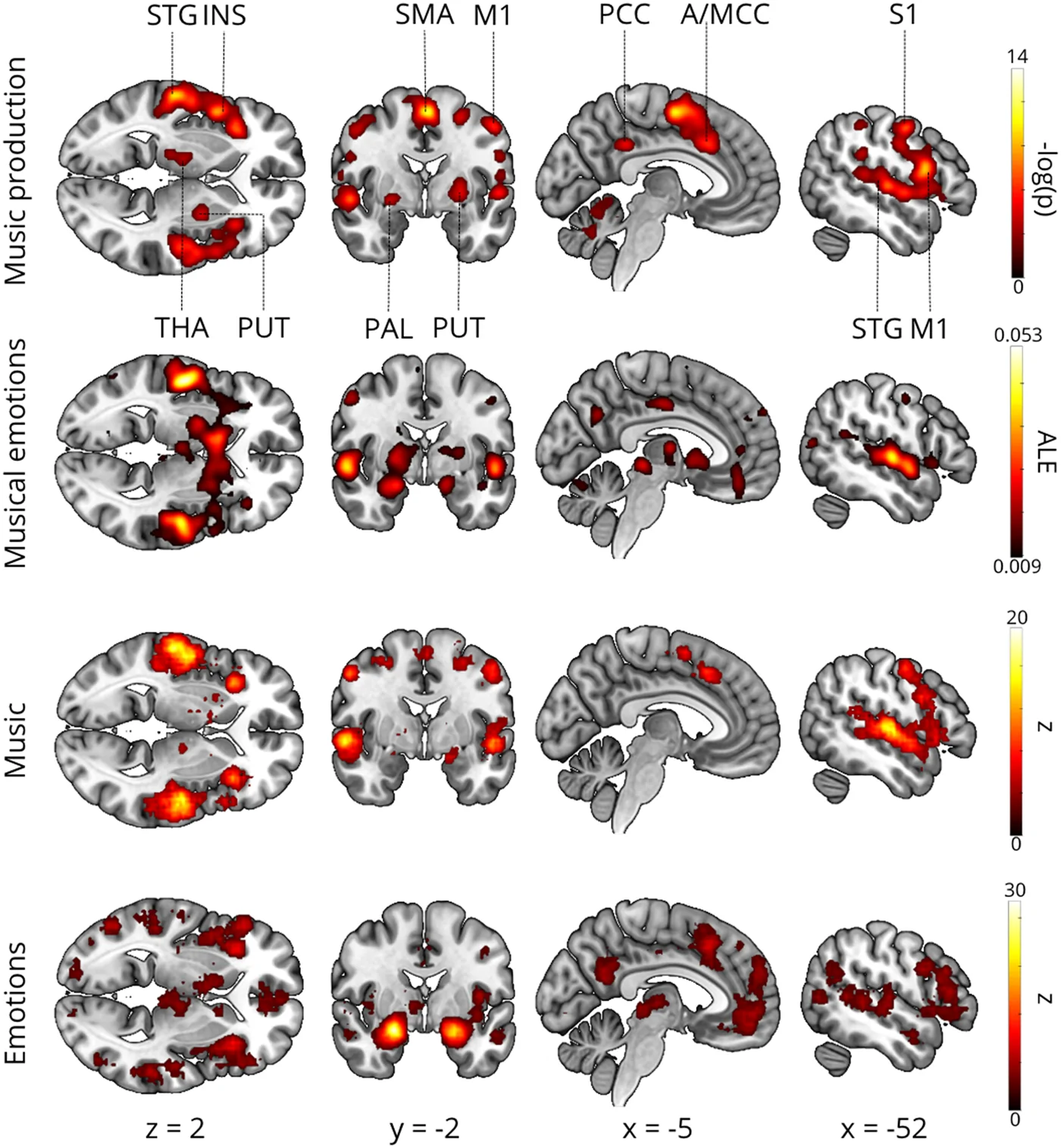
Brain regions involved in making music (music production) significantly overlap with those involved in processing of emotions and emotional expressions (emotions) across many cortical regions (e.g. midcingulate cortex, insula, SMA), but show minimal overlap in the canonical emotion structures (e.g. amygdala, the striatum). The data are cluster-corrected at *p* < 0.01 (family-wise error Montecarlo). INS, insula; PCC, posterior cingulate cortex; A/MCC, anterior and middle cingulate cortex; THA, thalamus; PUT, putamen; PAL, pallidum.

To inform the discussion of the brain basis of musical emotional communication, we conducted a meta-analysis (figure 1 and the supplementary material) of blood oxygenation level-dependent functional magnetic resonance imaging and positron emission tomography (PET) responses to producing music. Altogether, 40 studies, 848 subjects and 1727 activation foci were included in the analysis (supplementary material, table S1). Originally, our plan involved conducting a sub-meta-analysis of studies focusing on intended emotions during musical performance. However, because only a single study specifically investigated music production with emotional intent, all studies were pooled together. The resulting activation likelihood estimation (ALE) map was then compared with automatically generated meta-analytic maps for keywords ‘emotions’ and ‘music’ (15 October 2025) derived from the NeuroSynth database [39] as well as an ALE map from a recent meta-analysis on the brain basis of music-evoked emotions [16]. The NeuroSynth maps present the degree to which each voxel is consistently reported in studies using the respective term. By comparing the neural correlates associated with music production to those underlying emotions, music and music-evoked emotions, we can identify potential overlaps and address the affective components of music production at the neural level—an aspect that has been largely overlooked in the existing literature. This analysis (figure 1 and supplementary material file at https://osf.io/69qwv/) revealed that music production consistently engaged M1, supplementary motor area (SMA), bilateral superior temporal (STG) and Heschl's gyri, primary somatosensory cortex (S1), anterior insula, pallidum, anterior and midcingulate cortices. The effects in midcingulate cortices, insula, Heschl's gyrus and superior temporal gyrus, SMA and post-central and pre-central gyri were also observed for music in general. Of these effects, those in superior temporal cortices (Heschl's gyrus) overlapped with the music-evoked emotions ALE map.

**Figure 2.**
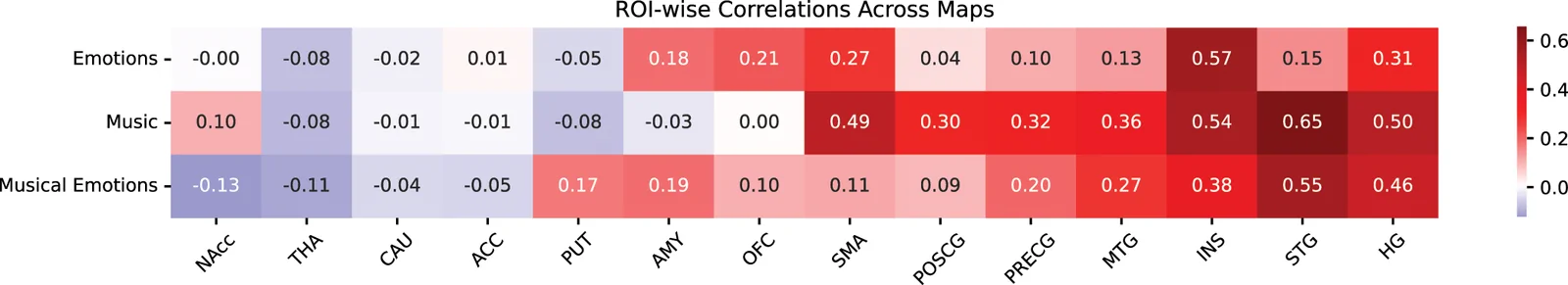
Regional overlap between brain regions involved in music making (the present meta-analysis), musical emotions [16], emotions in general (from NeuroSynth) and music in general (from NeuroSynth). ROI, region of interest; NAcc, nucleus accumbens; THA, thalamus; CAU, caudate nucleus; PUT, putamen; AMY, amygdala; OFC, orbitofrontal cortex; SMA, supplementary motor area; POSCG, postcentral gyrus; PRECG, precentral gyrus; MTG, middle temporal gyrus; INS, insula; STG, superior temporal gyrus; HG, Heschl's gyrus.

The meta-analytic maps for music production and emotions showed overlap in midcingulate cortices, M1, SMA, insula, thalamus and superior temporal cortices. Emotions further activated anterior cingulate cortex, amygdala and fusiform gyri, whereas these effects were absent for music making, while the amygdala activations were observed for emotions and music-evoked emotions. Functional coactivation analysis at the regional level revealed that the most consistent joint effects for music production, music, emotions and music-evoked emotions were observed in superior temporal cortices, insula and post- and pre-central gyrus (figure 2). These results suggest that music production engages partially similar brain networks as music-evoked emotions and in a more limited manner those involved in emotions. While paralimbic activations were observed for emotions as well as music-evoked emotions, limbic activations were markedly absent for the music production ALE analysis. Finally, music production uniquely engaged the primary auditory cortical core, belt and parabelt areas. In addition to encoding the acoustic features of the musical signal, these regions hold emotional representations of music as well as its musical and semantic (lyrical) information [18,40].

Together, these results confirm that music production robustly engages the somatomotor regions involved in the execution of the music as well as temporal cortical regions involved in auditory processing. The midcingulate regions probably reflect the motor and cognitive control, whereas the insular activations fall within the anterior affective and cognitive subdivisions of the insular cortex [41–43]. This indicates that music production robustly engages numerous components of the ‘extended’ emotion circuits in the brain, whereas activations in the core limbic areas are missing. While real-life music performance is often an emotionally engaging experience for the player, performing a scanner-compatible music production task without an audience may not in itself be an affective act at the neural level. This highlights that music production can be non-emotional in itself, even though the resulting musical expression causes emotions in others. This parallels data from other modalities with potential emotional communicative value. For example, when comparing brain responses to displaying versus observing facial expressions, both tasks robustly engage the somatomotor and paralimbic regions, whereas limbic activations are observed only when observing expressions [44]. Despite the communicative potential of music, emotion-circuit activation appears not to be a core feature of musical performance, at least in task paradigms centred on structured execution. Future neuroimaging work is needed to test how expressive intent and social–communicative contexts in musical performance modulate engagement of emotion-related neural systems.

## 5. Verbal modulation of musical emotions

Music is a significantly more complex signal than other emotion communication channels. Whereas facial and bodily emotional expressions rely on muscle configurations and kinetics, musical expression can be achieved through multiple acoustic features (pitch, loudness, rhythm, timbre, harmony…) and most notably, the lyrics. Singing appears in every culture, is predominantly done in groups and serves functions such as dance, healing, love and childcare [45]. Singing is also an effective means for musical emotional communication, because the brain processes singing as both language and music [40]. A large body of evidence suggests that affect can be conveyed effectively through semantics of written text and speech [46]. Lyrics are also used for communicating emotional qualities in songs and, for example, during the past 50 years the most common themes of the lyrics of popular music pertain to emotional topics such as relationships and love, sex, music, dance and partying and good times [47]. Lyrics are highly prevalent in many contemporary listening contexts, although instrumental genres such as EDM, jazz, classical and numerous non-Western musical traditions constitute important exceptions. Based on surveys of Western listeners, over 94% of people's favourite music contains lyrics [48], and when participants are asked to select their own favourite music for brain imaging experiments on musical affect, they almost invariably select music with lyrics [49,50]. Lyrics connect music with our innate allure for storytelling [51], allowing music to connect with meaningful narratives leading to collective resonance of emotions, and many popular songs considered deeply emotional masterpieces such as Bob Dylan's ‘Like a Rolling Stone' or The Beatles’ ‘Hey Jude’ would probably have substantially reduced emotional power if they had been released as instrumental versions.

Sentiment analysis has confirmed that a significant proportion of popular music lyrics is imbued with (primarily positive) emotion [52]. The semantic features of the lyrics are linked with the affective valence of music, complementing the emotional expression through the musical features so that the combination of lyrics and musical features allows the most accurate prediction of resultant affect [53]. Large-scale analysis also suggests a close link between harmony and lyrics, that is, presence of major/minor chords and concurrent emotional semantics of the lyrics [54], suggesting that musical and semantic features both contribute to the mood of a musical piece. When the emotion conveyed by the melody and the lyrics are incongruent (e.g. a happy melody with sad lyrics), melody tends to dominate the perceived emotion, suggesting that musical features may outweigh lyrical content when the two convey conflicting emotions [55]. The contribution of lyrics to the net emotion resulting from a musical piece, however, depends on the emotional qualities of the music. Happy music without lyrics evokes stronger positive emotions than happy music with lyrics, and at the neural level happy music without (but not with) lyrics robustly engages the limbic emotion circuits, whereas the opposite is true for sad music [56]. Similarly, behavioural studies have found that sad music paired with sad lyrics boosts the negative emotion induced by a song, whereas the same sad song played without lyrics was perceived as pleasant and had a positive effect on mood [57]. Other studies, in turn, have found evidence that lyrics are more fundamental in defining the affective qualities of sad versus happy music and even these results might be language-specific [55,58]. However, not all studies have been able to replicate these effects [59] and it is likely that lyrics and vocal expressions have partially independent effects on the evoked emotion, yet the vocal and musical expressions tend to dominate over semantics [60].

## 6. Audiovisual integration and musical emotions

As a live artform, music is inherently a multi-modal experience, because listeners not only hear but also see the performers. During musical performance, visual cues such as facial expressions and gestures both complement the musical performance as well as direct the audience's attention and engagement. The emotional qualities of music can be modulated by simultaneously occurring facial and bodily expressions of emotions, making it a particularly flexible tool for emotional communication. Audiovisual integration of emotions occurs across communicative channels such as facial expressions and the tone of voice [61], and these channels are also integrated for musical performances. Facial expressions that are congruent with the emotions expressed in the music amplify the resultant emotion in comparison with the music alone [62], and musical expressive cues have a larger impact on music-evoked emotions when the performance can be seen, indicating the additive effects of visual and acoustic channels in emotion elicitation [63]. Although some studies have failed to find evidence for such integration at subjective or psychophysiological levels [64], meta-analytic data support the integration of visual and auditory signals in the evaluation of expressiveness and aesthetic appreciation of musical performance [65]. Compared to recorded music, live music also leads to stronger responses in the amygdala and strengthens the coupling between listeners' brain activity, the acoustic features of the music and their emotional experiences, indicating amplified multimodal emotional integration [66].

Performers' facial and other non-verbal expressions complement music, influencing the interpretation of both musical and affective qualities of the performance [67]. Facial movements associated with emotions during speech are retained during singing [68], and the visual expressive cues amplify the emotional content of the music [69]. Facial movements during singing often exceed what would be required for sound production alone and therefore probably contribute to emotional expressivity. In line with this, observers can more accurately infer the emotional meaning from visual and audiovisual than from audio-only song recordings, highlighting the importance of facial motion in communicating emotions through musical performance [70]. Expressive body movements during musical performance reflect a musician's internal states and serve as a channel for emotional communication. Observers rely heavily on visual cues to infer expressiveness, with non-musicians often identifying expressive intent more accurately through sight than sound [71]. Specific emotions—such as happiness or anger—can be distinguished from musicians' body movements alone [72]. The kinematic features that convey expressive intent, including movement amplitude, speed and smoothness, are particularly evident in body parts not directly involved in sound production, such as the head and upper torso [73]. Listeners can also perceive emotional dimensions—valence, activity and power—from conductors' point-light gestures alone, further demonstrating that expressive movement communicates nuanced affective content even in the absence of sound [74].

People are also not merely passive listeners but move to music and attend to visual and bodily cues in musical performance, which contribute to the communication of musical emotion. Emotional qualities of music are mirrored in bodily movement, highlighting a close coupling of movement and musical emotion expression. Adult observers can recognize emotions like anger, fear and joy above chance from movements of professional dancers [75], while the spontaneous movements of untrained listeners dancing to music reflect its emotional qualities in systematic ways [76], and the motions induced by the music in the listeners can be predicted by rhythm, timbre and dynamics of the music [77]. The perceptual skills required to detect musical features closely tied to movement, such as beat and tempo, are present early in development, and children not only recognize but also spontaneously move to music from a young age [78]. Musical rhythm also structures social attention and parent–infant interaction. During infant-directed singing, carers' expressive facial cues are rhythmically organized around the musical beat, and infants preferentially time their gaze to carers' eyes at those same moments [79].

Children as young as five can already decode at least some emotions from dance movements [80], suggesting that bodily movement is an early-emerging channel for musical emotion communication. This early coupling of perception and action provides the foundation for later sensitivity to rhythmic and dynamic properties of music that elicit an urge to move. Research on ‘groove’ indicates that this urge is modulated by rhythmic complexity—typically strongest at moderate levels of syncopation—and that this appears robust across cultures [81]. Features that tend to be perceived as expressing positive emotions such as major mode tend to also boost the urge to move and the sense of groove, and elicit bodily sensation in the limbs possibly related to the urge to move [82,83]. Thus, the expression, perception and induction of musical emotions are deeply intertwined with bodily movement from early development onwards, providing a foundation for collective synchronization and social interaction that characterizes much of human musical activities.

## 7. Social musical emotions

Finally, we turn to the question of what the ultimate function of musical emotional communication is. One prominent explanation is that music offers a unique way for promoting social connections by allowing the collective synchronization of emotions and moods of large groups on a scale far exceeding what would be possible via other expressive channels. Historically, this synchronizing function unfolded primarily in co-present settings: although individuals have always been able to make music in solitude, the now-common practice of listening alone to performances by absent others became possible only after the invention of analogue sound recording in 1877 by Thomas Edison and has expanded dramatically with the advent of digital recording and global online music distribution. Cross-cultural studies have revealed that musical universals are not limited to pitch- and rhythm-related musical features, but also encompass group musical performance in social contexts [84]. Similarly, a large-scale analysis of audio recordings from more than 1000 societies and ethnographic texts from over 60 corpora suggests that music making (singing) in groups is far more common than solitary musical expression [85]. Remarkably, listeners from diverse cultural backgrounds can reliably recognize dance songs, lullabies and healing songs even without prior exposure [86]. This suggests that features like tempo, rhythmic and melodic complexity and dynamics are systematically tied to these social functions and draw on broadly recognizable expressive cues.

Given that emotional engagement increases neural entrainment across individuals [87,88], musical emotion communication might harness this same mechanism to promote interpersonal synchronization and engagement in social rituals. Anthropologists and psychologists alike have proposed that synchronized action in rituals may enhance group cohesion, promoting cooperation and tightening social bonds, because they bring masses of people together in coordinated motions and minds [89,90]. Musical synchrony in ritual contexts may amplify these processes by inducing shared emotion, thereby strengthening group affiliation and credibly signalling cohesion to outsiders [12,13]. Increased neural coupling (as indexed by intersubject synchronization (ISC) across individuals) is observed particularly during affect-laden episodes of speech and cinema [87,88,91], and the degree of ISC is indicative of psychological attunement of the individuals [92]. When listening to affective music, such synchronization peaks in the emotion circuits including the amygdala, thalamus and caudate nucleus, which highlights the capacity of musical emotions to ‘tune in’ different individuals [93].

This is supported by findings that body movement plays a central role in coordinating musical actions within ensembles [94] and may also shape the emotional expressiveness of music through interpersonal coordination among musicians [95]. Coordinated body sway among performers serves as a medium for greater information flow during expressive versus non-expressive performances, suggesting that it reflects joint emotional expression and is associated with higher perceived emotional intensity by listeners. During extremely energized music-evoked emotions such as excitement during a heavy metal concert, the collective behaviour can self-organize from disordered gas-like states in the mosh pit into ordered vortex-like states of the circle pits [96], providing an effective means for mass synchronization.

Musical performance can also have many intraindividual consequences for the performer, ranging from mood regulation, stress reduction and intrinsically rewarding experiences such as flow or pleasure to performance anxiety, perfectionism and elevated emotional strain [97]. Performing can elicit emotions such as exhilaration, flow and heightened arousal. These experiences may be embedded in social contexts involving co-performers and audiences [98], where shared attention, evaluation and coordination amplify emotional intensity and confer meaning on the event. Solitary playing may also have similar effects that may arise from engagement, absorption or the rewarding nature of musical activity. Yet, even solitary performance can involve the internal simulation of a listener or imagined audience or co-performers [99], thereby possibly engaging expressive systems that primarily serve interpersonal communication. From this perspective, some of the intra-individual effects of music making can be understood as consequences of activating communicative systems that are typically realized in social interaction.

A large bulk of evidence suggests that interpersonal synchrony is an important means for social bonding and signifying alliances, a process which is thought to be mediated through the endogenous opioid system [100–102]. PET imaging studies show that pleasurable music is capable of engaging not only the dopamine system central for reward and incentive motivation [103], but also the endogenous opioid system [49], which has been implicated in both positive and negative emotions as well as affiliative behaviour and social bonding [104]. Mu-opioid receptors are also expressed in the vestibular afferent neurons [105,106], and in humans, head nodding while listening to music increases pain threshold—an indirect proxy for endogenous opioid release. Accordingly, shared rhythmicity and entrainment of actions, breathing and autonomic activation while singing or playing can provide a rhythmic framework facilitating synchrony [107], potentially engaging opioid-mediated social bonding. Such bonding functions may also operate independently of the actual mutual music making. Merely listening to music can reduce feelings of loneliness and it has been proposed that music might act as a ‘social surrogate’; thus music may not only be used for establishing social bonds but may simulate social contact, perhaps because it is perceived as expressing the emotions and intentions of another mind [108].

## 8. Conclusions

**Figure 3.**
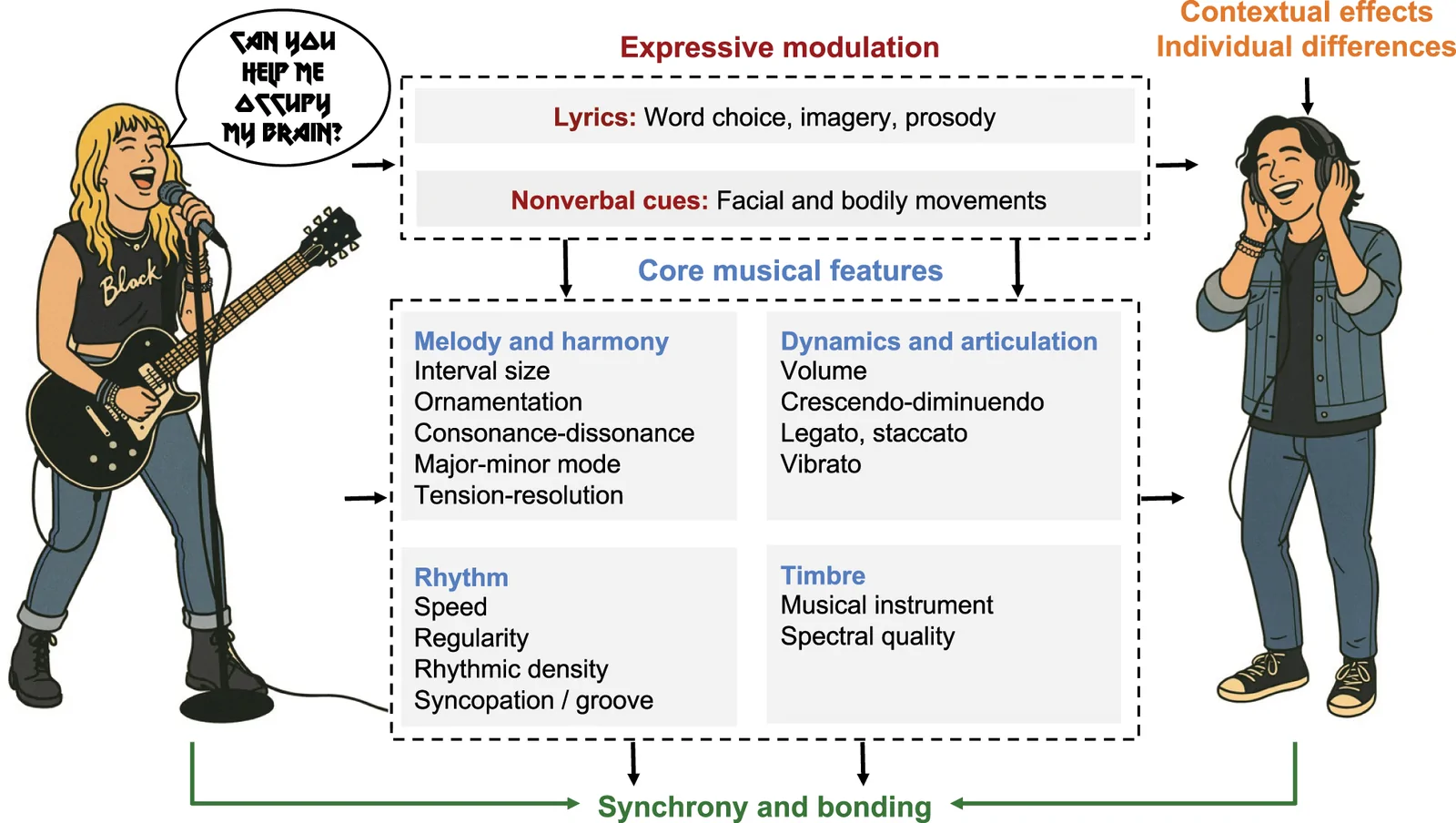
Emotional communication channels in music. The emotional tone of the music is primarily determined by the core musical features, which can, however, be altered by concomitant non-verbal and verbal expressive channels.

Although laboratory music production tasks do not consistently engage emotion circuits, emotional qualities are inherently embedded into music, and musical communication is a powerful way of communicating emotion for establishing and maintaining social bonds (figure 3). This is accomplished by a set of core musical features that generate the overall affective tone of the music, based on shared acoustic code between musical and vocal expressions [6], which is complemented by learned associations between musical emotions and features such as musical expressive cues and harmony [27–29]. This core musical expression can be further complemented by lyrical and non-verbal cues presented simultaneously with the musical performance, although several studies suggest that musical features often play a primary role in determining perceived emotion. Owing to this multichannel nature of emotional communication, music offers a unique and rich means of transmitting affect and connecting minds across cultural and situational contexts.

## Supplementary Material



## Data Availability

Meta-analytic database and unthresholded result files are available on OSF at [109]. Supplementary material is available online [110].

## Footnote

^1^
We recognize that today these boundaries can be overcome through digital communication and mass media, and the presented distinctions apply to emotional communication without technological aids.

## Theme

One contribution of 19 to a theme issue ‘Mechanisms, development, phylogeny and functions of emotional expressions’.

## References

[1] Trainor LJ . 2015 The origins of music in auditory scene analysis and the roles of evolution and culture in musical creation. Phil. Trans. R. Soc. B 370, 20140089. (doi:10.1098/rstb.2014.0089) PMC4321130 25646512

[2] Schäfer T , Sedlmeier P. 2009 From the functions of music to music preference. Psychol. Music 37, 279–300. (doi:10.1177/0305735608097247)

[3] Tarr B , Launay J, Dunbar RIM. 2014 Music and social bonding: ‘self-other’ merging and neurohormonal mechanisms. Front. Psychol. 5, 1096. (doi:10.3389/fpsyg.2014.01096) PMC4179700 25324805

[4] Tarrant M , North AC, Hargreaves DJ. 2000 English and American adolescents' reasons for listening to music. Psychol. Music 28, 166–173. (doi:10.1177/0305735600282005)

[5] Kullgren J , Solway E, Roberts S, Howell J, Singer D, Box N, Smith E, Hutchens L. 2024 National poll on healthy aging: sound of music. Ann Arbor, MI: Institute for Healthcare Policy & Innovation (IHPI). (doi:10.7302/22174)

[6] Juslin PN , Laukka P. 2004 Expression, perception, and induction of musical emotions: a review and a questionnaire study of everyday listening. J. New Music Res. 33, 217–238. (doi:10.1080/0929821042000317813)

[7] Lindström E , Juslin PN, Bresin R, Williamon A. 2003 ‘Expressivity comes from within your soul': a questionnaire study of music students’ perspectives on expressivity. Res. Stud. Music Educ. 20, 23–47. (doi:10.1177/1321103X030200010201)

[8] Nummenmaa L , Saarimäki H. 2017 Emotions as discrete patterns of systemic activity. Neurosci. Lett. 693, 3–8. (doi:10.1016/j.neulet.2017.07.012) 28705730

[9] Nummenmaa L , Glerean E, Hari R, Hietanen JK. 2014 Bodily maps of emotions. Proc. Natl Acad. Sci. USA 111, 646–651. (doi:10.1073/pnas.1321664111) PMC3896150 24379370

[10] Adolphs R . 2002 Recognizing emotion from facial expressions: psychological and neurological mechanisms. Behav. Cogn. Neurosci. Rev. 1, 21–62. (doi:10.1177/1534582302001001003) 17715585

[11] Fritz T , Jentschke S, Gosselin N, Sammler D, Peretz I, Turner R, Friederici AD, Koelsch S. 2009 Universal recognition of three basic emotions in music. Curr. Biol. 19, 573–576. (doi:10.1016/j.cub.2009.02.058) 19303300

[12] Savage PE , Loui P, Tarr B, Schachner A, Glowacki L, Mithen S, Fitch WT. 2020 Music as a coevolved system for social bonding. Behav. Brain Sci. 44, e59. (doi:10.1017/S0140525X20000333) 32814608

[13] Mehr SA , Krasnow MM, Bryant GA, Hagen EH. 2020 Origins of music in credible signalling. Behav. Brain Sci. 44, e60. (doi:10.1017/S0140525X20000345) PMC7907251 32843107

[14] Cowen AS , Fang X, Sauter D, Keltner D. 2020 What music makes us feel: at least 13 dimensions organize subjective experiences associated with music across different cultures. Proc. Natl Acad. Sci. USA 117, 1924. (doi:10.1073/pnas.1910704117) PMC6995018 31907316

[15] Juslin PN , Laukka P. 2003 Communication of emotions in vocal expression and music performance: different channels, same code? Psychol. Bull. 129, 770–814. (doi:10.1037/0033-2909.129.5.770) 12956543

[16] Nummenmaa L , Putkinen V, Sams M. 2021 Social pleasures of music. Curr. Opin. Behav. Sci. 39, 196–202. (doi:10.1016/j.cobeha.2021.03.026)

[17] Koelsch S . 2020 A coordinate-based meta-analysis of music-evoked emotions. Neuroimage 223, 117350. (doi:10.1016/j.neuroimage.2020.117350) 32898679

[18] Putkinen V *et al.* 2021 Decoding music-evoked emotions in the auditory and motor cortex. Cereb. Cortex 31, 2549–2560. (doi:10.1093/cercor/bhaa373) 33367590

[19] Koelsch S , Cheung VKM, Jentschke S, Haynes J-D. 2021 Neocortical substrates of feelings evoked with music in the ACC, insula, and somatosensory cortex. Sci. Rep. 11, 10119. (doi:10.1038/s41598-021-89405-y) PMC8115666 33980876

[20] Putkinen V , Zhou X, Gan X, Yang L, Becker B, Sams M, Nummenmaa L. 2024 Bodily maps of musical sensations across cultures. Proc. Natl Acad. Sci. USA 121, e2308859121. (doi:10.1073/pnas.2308859121) PMC10835118 38271338

[21] Eerola T , Vuoskoski JK. 2013 A review of music and emotion studies: approaches, emotion models, and stimuli. Music Percept. Interdiscip. J. 30, 307. (doi:10.1525/mp.2012.30.3.307)

[22] Juslin PN . 2012 Emotion in music performance, Oxford, UK: Oxford University Press.

[23] Schindler I , Hosoya G, Menninghaus W, Beermann U, Wagner V, Eid M, Scherer KR. 2017 Measuring aesthetic emotions: a review of the literature and a new assessment tool. PLoS ONE 12, e0178899. (doi:10.1371/journal.pone.0178899) PMC5459466 28582467

[24] Balkwill L-L , Thompson WF. 1999 A cross-cultural investigation of the perception of emotion in music: psychophysical and cultural cues. Music Percept. 17, 43–64. (doi:10.2307/40285811)

[25] Laukka P , Eerola T, Thingujam NS, Yamasaki T, Beller G. 2013 Universal and culture-specific factors in the recognition and performance of musical affect expressions. Emotion 13, 434–449. (doi:10.1037/a0031388) 23398579

[26] Meyer LB . 1956 Emotion and meaning in music, Chicago, IL: The University of Chicago Press.

[27] Bhatara A , Tirovolas AK, Duan LM, Levy B, Levitin DJ. 2011 Perception of emotional expression in musical performance. J. Exp. Psychol. Hum. Percept. Perform. 37, 921–934. (doi:10.1037/a0021922) 21261418

[28] Athanasopoulos G , Eerola T, Lahdelma I, Kaliakatsos-Papakostas M. 2021 Harmonic organisation conveys both universal and culture-specific cues for emotional expression in music. PLoS ONE 16, e0244964. (doi:10.1371/journal.pone.0244964) PMC7806179 33439887

[29] Smit EA , Milne AJ, Sarvasy HS, Dean RT. 2022 Emotional responses in Papua New Guinea show negligible evidence for a universal effect of major versus minor music. PLoS ONE 17, e0269597. (doi:10.1371/journal.pone.0269597) PMC9242494 35767551

[30] Di Stefano N , Spence C. 2022 Roughness perception: a multisensory/crossmodal perspective. Atten. Percept. Psychophys. 84, 2087–2114. (doi:10.3758/s13414-022-02550-y) PMC9481510 36028614

[31] Meeren HKM , van Heijnsbergen CCRJ, de Gelder B. 2005 Rapid perceptual integration of facial expression and emotional body language. Proc. Natl Acad. Sci. USA 102, 16518–16523. (doi:10.1073/pnas.0507650102) PMC1283446 16260734

[32] Nordström H , Laukka P. 2019 The time course of emotion recognition in speech and music. J. Acoust. Soc. Am. 145, 3058. (doi:10.1121/1.5108601) 31153307

[33] Vuust P , Heggli OA, Friston KJ, Kringelbach ML. 2022 Music in the brain. Nat. Rev. Neurosci. 23, 287–305. (doi:10.1038/s41583-022-00578-5) 35352057

[34] Salimpoor VN , Zald DH, Zatorre RJ, Dagher A, McIntosh AR. 2015 Predictions and the brain: how musical sounds become rewarding. Trends Cogn. Sci. 19, 86–91. (doi:10.1016/j.tics.2014.12.001) 25534332

[35] Calvo MG , Gutiérrez-García A, Fernández-Martín A, Nummenmaa L. 2014 Recognition of facial expressions of emotion is related to their frequency in everyday life. J. Nonverbal Behav. 38, 549–567. (doi:10.1007/s10919-014-0191-3)

[36] Sherstinova T . 2015Some observations on everyday singing behaviour based on long-term audio recordings. In Communications in computer and information science, vol. 561 (eds P Eismont, N Konstantinova), pp. 88–100. Cham, Switzerland: Springer International Publishing.

[37] Dean B . 2021 Spontaneous singing in early childhood: an examination of young children's singing at home. Res. Stud. Music Educ. 43, 434–450. (doi:10.1177/1321103X20924139)

[38] Hari R , Kujala MV. 2009 Brain basis of human social interaction: from concepts to brain imaging. Physiol. Rev. 89, 453–479. (doi:10.1152/physrev.00041.2007) 19342612

[39] Yarkoni T , Poldrack RA, Nichols TE, Van Essen DC, Wager TD. 2011 NeuroSynth: a new platform for large-scale automated synthesis of human functional neuroimaging data. Front. Neuroinf. (doi:10.3389/conf.fninf.2011.08.00058) PMC3146590 21706013

[40] Salmi J *et al.* 2017 Distributed neural signatures of natural audiovisual speech and music in the human auditory cortex. Neuroimage 157, 108–117. (doi:10.1016/j.neuroimage.2016.12.005) 27932074

[41] Uddin LQ , Nomi JS, Hébert-Seropian B, Ghaziri J, Boucher O. 2017 Structure and function of the human insula. J. Clin. Neurophysiol. 34, 300–306. (doi:10.1097/WNP.0000000000000377) PMC6032992 28644199

[42] Kurth F , Zilles K, Fox PT, Laird AR, Eickhoff SB. 2010 A link between the systems: functional differentiation and integration within the human insula revealed by meta-analysis. Brain Struct. Funct. 214, 519–534. (doi:10.1007/s00429-010-0255-z) PMC4801482 20512376

[43] Kwon M , Bo K, Botvinik-Nezer R, Kragel PA, Van Oudenhove L, Wager TD, The Affective Neuroimaging Consortium. 2025 Convergent and selective representations of pain, appetitive processes, aversive processes, and cognitive control in the insula. bioRxiv. (doi:10.1101/2025.02.18.638889) PMC13254212 41980935

[44] Volynets S , Smirnov D, Saarimäki H, Nummenmaa L. 2020 Statistical pattern recognition reveals shared neural signatures for displaying and recognizing specific facial expressions. Soc. Cogn. Affect. Neurosci. 15, 803–813. (doi:10.1093/scan/nsaa110) PMC7543934 33007782

[45] Mehr SA *et al.* 2019 Universality and diversity in human song. Science 366, eaax0868. (doi:10.1126/science.aax0868) PMC7001657 31753969

[46] Kotz SA , Paulmann S. 2011 Emotion, language, and the brain. Lang. Linguist. Compass 5, 108–125. (doi:10.1111/j.1749-818X.2010.00267.x)

[47] Christenson PG , de Haan-Rietdijk S, Roberts DF, ter Bogt TFM. 2019 What has America been singing about? Trends in themes in the U.S. top-40 songs: 1960–2010. Psychol. Music 47, 194–212. (doi:10.1177/0305735617748205)

[48] Conrad F , Corey J, Goldstein S, Ostrow J, Sadowsky M. 2019 Extreme re-listening: songs people love… and continue to love. Psychol. Music 47, 158–172. (doi:10.1177/0305735617751050)

[49] Putkinen V , Seppälä K, Harju H, Hirvonen J, Karlsson HK, Nummenmaa L. 2025 Pleasurable music activates cerebral µ-opioid receptors: a combined PET-fMRI study. Eur. J. Nucl. Med. Mol. Imaging 52, 3540. (doi:10.1007/s00259-025-07232-z) PMC12316753 40183950

[50] Putkinen V , Hahn A, Tuisku J, Harju H, Seppälä K, Kirjavainen AK, Rajander J, Hirvonen J, Nummenmaa L. 2025 Cerebral glucose utilisation during musical emotions: a multimodal functional pet/MRI study. bioRxiv. (doi:10.1101/2025.08.28.672829) 42250833

[51] Zunshine L . 2006 Why we read fiction: theory of mind and the novel, Columbus, OH: Ohio State University Press.

[52] Napier K , Shamir L. 2018 Quantitative sentiment analysis of lyrics in popular music. J. Popul. Music Stud. 30, 161–176. (doi:10.1525/jpms.2018.300411)

[53] Laurier C , Grivolla J, Herrera P. 2008 Multimodal music mood classification using audio and lyrics. In 2008 Seventh Int. Conf. Machine Learning and Applications, San Diego, CA, USA, pp. 688–693. Piscataway, NJ: IEEE. (doi:10.1109/ICMLA.2008.96)

[54] Kolchinsky A , Dhande N, Park K, Ahn Y-Y. 2017 The minor fall, the major lift: inferring emotional valence of musical chords through lyrics. R. Soc. Open Sci. 4, 170952. (doi:10.1098/rsos.170952) PMC5717663 29291089

[55] Ali SO , Peynircioğlu ZF. 2006 Songs and emotions: are lyrics and melodies equal partners? Psychol. Music 34, 511–534. (doi:10.1177/0305735606067168)

[56] Brattico E , Alluri V, Bogert B, Jacobsen T, Vartiainen N, Nieminen S, Tervaniemi M. 2011 A functional MRI study of happy and sad emotions in music with and without lyrics. Front. Psychol. 2, 308. (doi:10.3389/fpsyg.2011.00308) PMC3227856 22144968

[57] Stratton VN , Zalanowski AH. 1994 Affective impact of music vs. lyrics. Empir. Stud. Arts 12, 173–184. (doi:10.2190/35T0-U4DT-N09Q-LQHW)

[58] Barradas GT , Sakka LS. 2022 When words matter: a cross-cultural perspective on lyrics and their relationship to musical emotions. Psychol. Music 50, 650–669. (doi:10.1177/03057356211013390)

[59] Sousou SD . 1997 Effects of melody and lyrics on mood and memory. Percept. Mot. Skills 85, 31–40. (doi:10.2466/pms.1997.85.1.31) 9293553

[60] Pond N . 2025 Comparing the emotional effects of semantic content and vocal expression of lyrics. Psychol. Music. 54, 514–528. (doi:10.1177/03057356251334142)

[61] de Gelder B , Vroomen J. 2000 The perception of emotions by ear and by eye. Cogn. Emot. 14, 289–311. (doi:10.1080/026999300378824)

[62] Pan F , Zhang L, Ou Y, Zhang X. 2019 The audio-visual integration effect on music emotion: behavioural and physiological evidence. PLoS ONE 14, e0217040. (doi:10.1371/journal.pone.0217040) PMC6542535 31145745

[63] Vines BW , Krumhansl CL, Wanderley MM, Dalca IM, Levitin DJ. 2011 Music to my eyes: cross-modal interactions in the perception of emotions in musical performance. Cognition 118, 157–170. (doi:10.1016/j.cognition.2010.11.010) 21146164

[64] Vuoskoski JK , Gatti E, Spence C, Clarke EF. 2016 Do visual cues intensify the emotional responses evoked by musical performance? A psychophysiological investigation. Psychomusicology 26, 179–188. (doi:10.1037/pmu0000142)

[65] Platz F , Kopiez R. 2012 When the eye listens: a meta-analysis of how audio-visual presentation enhances the appreciation of music performance. Music Percept. 30, 71–83. (doi:10.1525/mp.2012.30.1.71)

[66] Trost W , Trevor C, Fernandez N, Steiner F, Frühholz S. 2024 Live music stimulates the affective brain and emotionally entrains listeners in real time. Proc. Natl Acad. Sci. USA 121, e2316306121. (doi:10.1073/pnas.2316306121) PMC10927510 38408255

[67] Thompson WF , Graham P, Russo FA. 2005 Seeing music performance: visual influences on perception and experience. Semiotica 156, 203–227. (doi:10.1515/semi.2005.2005.156.203)

[68] Livingstone SR , Thompson WF, Russo FA. 2009 Facial expressions and emotional singing: a study of perception and production with motion capture and electromyography. Music Percept. 26, 475–488. (doi:10.1525/mp.2009.26.5.475)

[69] Thompson WF , Russo FA, Quinto L. 2008 Audio-visual integration of emotional cues in song. Cogn. Emot. 22, 1457–1470. (doi:10.1080/02699930701813974)

[70] Livingstone SR , Thompson WF, Wanderley MM, Palmer C. 2015 Common cues to emotion in the dynamic facial expressions of speech and song. Q. J. Exp. Psychol. (Hove) 68, 952–970. (doi:10.1080/17470218.2014.971034) PMC4440649 25424388

[71] Davidson JW . 1993 Visual perception of performance manner in the movements of solo musicians. Psychol. Music 21, 103–113. (doi:10.1177/030573569302100201)

[72] Dahl S , Friberg A. 2007 Visual perception of expressiveness in musicians' body movements. Music Percept. 24, 433–454. (doi:10.1525/mp.2007.24.5.433)

[73] Thompson MR , Luck G. 2012 Exploring relationships between pianists' body movements, their expressive intentions, and structural elements of the music. Music. Sci. 16, 19–40. (doi:10.1177/1029864911423457)

[74] Luck G , Toiviainen P, Thompson MR. 2010 Perception of expression in conductors' gestures: a continuous response study. Music Percept. 28, 47–57. (doi:10.1525/mp.2010.28.1.47)

[75] Camurri A , Lagerlöf I, Volpe G. 2003 Recognizing emotion from dance movement: comparison of spectator recognition and automated techniques. Int. J. Hum. Comput. Stud. 59, 213–225. (doi:10.1016/S1071-5819(03)00050-8)

[76] Van Dyck E , Maes P-J, Hargreaves J, Lesaffre M, Leman M. 2013 Expressing induced emotions through free dance movement. J. Nonverbal Behav. 37, 175–190. (doi:10.1007/s10919-013-0153-1)

[77] Burger B , Thompson MR, Luck G, Saarikallio S, Toiviainen P. 2013 Influences of rhythm- and timbre-related musical features on characteristics of music-induced movement. Front. Psychol. 4, 183. (doi:10.3389/fpsyg.2013.00183) PMC3624091 23641220

[78] Zentner M , Eerola T. 2010 Rhythmic engagement with music in infancy. Proc. Natl Acad. Sci. USA 107, 5768–5773. (doi:10.1073/pnas.1000121107) PMC2851927 20231438

[79] Lense MD , Shultz S, Astésano C, Jones W. 2022 Music of infant-directed singing entrains infants' social visual behaviour. Proc. Natl Acad. Sci. USA 119, e2116967119. (doi:10.1073/pnas.2116967119) PMC9659341 36322755

[80] Boone RT , Cunningham JG. 1998 Children's decoding of emotion in expressive body movement: the development of cue attunement. Dev. Psychol. 34, 1007–1016. (doi:10.1037/0012-1649.34.5.1007) 9779746

[81] Witek MAG , Clarke EF, Wallentin M, Kringelbach ML, Vuust P. 2014 Syncopation, body-movement and pleasure in groove music. PLoS ONE 9, e94446. (doi:10.1371/journal.pone.0094446) PMC3989225 24740381

[82] Matthews TE , Witek MAG, Heggli OA, Penhune VB, Vuust P. 2019 The sensation of groove is affected by the interaction of rhythmic and harmonic complexity. PLoS ONE 14, e0204539. (doi:10.1371/journal.pone.0204539) PMC6328141 30629596

[83] Kawase S . 2024 Is happier music groovier? The influence of emotional characteristics of musical chord progressions on groove. Psychol. Res. 88, 438–448. (doi:10.1007/s00426-023-01869-x) PMC10858120 37615754

[84] Savage PE , Brown S, Sakai E, Currie TE. 2015 Statistical universals reveal the structures and functions of human music. Proc. Natl Acad. Sci. USA 112, 8987–8992. (doi:10.1073/pnas.1414495112) PMC4517223 26124105

[85] Shilton D , Passmore S, Savage PE. 2023 Group singing is globally dominant and associated with social context. R. Soc. Open Sci. 10, 230562. (doi:10.1098/rsos.230562) PMC10480695 37680502

[86] Mehr SA , Singh M, York H, Glowacki L, Krasnow MM. 2018 Form and function in human song. Curr. Biol. 28, 356–368.e5. (doi:10.1016/j.cub.2017.12.042) PMC5805477 29395919

[87] Nummenmaa L , Glerean E, Viinikainen M, Jääskeläinen IP, Hari R, Sams M. 2012 Emotions promote social interaction by synchronizing brain activity across individuals. Proc. Natl Acad. Sci. USA 109, 9599–9604. (doi:10.1073/pnas.1206095109) PMC3386135 22623534

[88] Smirnov D , Saarimäki H, Glerean E, Hari R, Sams M, Nummenmaa L. 2019 Emotions amplify speaker–listener neural alignment. Hum. Brain Mapp. 40, 4777–4788. (doi:10.1002/hbm.24736) PMC6865790 31400052

[89] Lakin JL , Jefferis VE, Cheng CM, Chartrand TL. 2003 The chameleon effect as social glue: evidence for the evolutionary significance of non-conscious mimicry. J. Nonverbal Behav. 27, 145–162. (doi:10.1023/A:1025389814290)

[90] Dunbar RIM . 2003 The social brain: mind, language, and society in evolutionary perspective. Annu. Rev. Anthropol. 32, 163–181. (doi:10.1146/annurev.anthro.32.061002.093158)

[91] Nummenmaa L , Saarimäki H, Glerean E, Gotsopoulos A, Jääskeläinen IP, Hari R, Sams M. 2014 Emotional speech synchronizes brains across listeners and engages large-scale dynamic brain networks. Neuroimage 102, 498–509. (doi:10.1016/j.neuroimage.2014.07.063) PMC4229500 25128711

[92] Lahnakoski JM , Glerean E, Jääskeläinen IP, Hyönä J, Hari R, Sams M, Nummenmaa L. 2014 Synchronous brain activity across individuals underlies shared psychological perspectives. NeuroImage 100, 316–324. (doi:10.1016/j.neuroimage.2014.06.022) PMC4153812 24936687

[93] Trost W , Frühholz S, Cochrane T, Cojan Y, Vuilleumier P. 2015 Temporal dynamics of musical emotions examined through intersubject synchrony of brain activity. Soc. Cogn. Affect. Neurosci. 10, 1705–1721. (doi:10.1093/scan/nsv060) PMC4666110 25994970

[94] Chang A , Livingstone SR, Bosnyak DJ, Trainor LJ. 2017 Body sway reflects leadership in joint music performance. Proc. Natl Acad. Sci. USA 114, E4134–E4141. (doi:10.1073/pnas.1617657114) PMC5448222 28484007

[95] Chang A , Kragness HE, Livingstone SR, Bosnyak DJ, Trainor LJ. 2019 Body sway reflects joint emotional expression in music ensemble performance. Sci. Rep. 9, 205. (doi:10.1038/s41598-018-36358-4) PMC6338747 30659220

[96] Silverberg JL , Bierbaum M, Sethna JP, Cohen I. 2013 Collective motion of humans in mosh and circle pits at heavy metal concerts. Phys. Rev. Lett. 110, 228701. (doi:10.1103/PhysRevLett.110.228701) 23767754

[97] Woody RH , McPherson GE. 2010Emotion and motivation in the lives of performers. In PN Juslin, JA Sloboda (Eds.), Handbook of music and emotion: Theory, research, applications, pp. 401–424. Oxford University Press.

[98] Lamont A . 2012 Emotion, engagement and meaning in strong experiences of music performance. Psychology of Music, 40, 574–594.

[99] Van Zijl AGW , Sloboda J. 2011 Performers' experienced emotions in the construction of expressive musical performance: An exploratory investigation. Psychology of Music, 39, 196–219.

[100] Machin A , Dunbar RIM. 2011 The brain opioid theory of social attachment: a review of the evidence. Behaviour 148, 985–1025. (doi:10.1163/000579511X596624)

[101] Chartrand TL , Bargh JA. 1999 The chameleon effect: the perception–behaviour link and social interaction. J. Pers. Soc. Psychol. 76, 893–910. (doi:10.1037/0022-3514.76.6.893) 10402679

[102] Manninen S *et al.* 2017 Social laughter triggers endogenous opioid release in humans. J. Neurosci. 37, 6125–6131. (doi:10.1523/JNEUROSCI.0688-16.2017) PMC6596504 28536272

[103] Salimpoor VN , Benovoy M, Larcher K, Dagher A, Zatorre RJ. 2011 Anatomically distinct dopamine release during anticipation and experience of peak emotion to music. Nat. Neurosci. 14, 257–U355. (doi:10.1038/nn.2726) 21217764

[104] Nummenmaa L , Tuominen LJ. 2018 Opioid system and human emotions. Br. J. Pharmacol. 175, 2737–2749. (doi:10.1111/bph.13812) PMC6016642 28394427

[105] Popper P , Cristobal R, Wackym PA. 2004 Expression and distribution of mu opioid receptors in the inner ear of the rat. Neuroscience 129, 225–233. (doi:10.1016/j.neuroscience.2004.08.008) 15489044

[106] Jongkamonwiwat N , Phansuwan-Pujito P, Sarapoke P, Chetsawang B, Casalotti SO, Forge A, Dodson H, Govitrapong P. 2003 The presence of opioid receptors in rat inner ear. Hear. Res. 181, 85–93. (doi:10.1016/S0378-5955(03)00175-8) 12855366

[107] Dunbar RIM , Pearce E, Tarr B, Makdani A, Bamford J, Smith S, McGlone F. 2021 Cochlear SGN neurons elevate pain thresholds in response to music. Sci. Rep. 11, 14547. (doi:10.1038/s41598-021-93969-0) PMC8282857 34267302

[108] Schäfer K , Saarikallio S, Eerola T. 2020 Music may reduce loneliness and act as social surrogate for a friend: evidence from an experimental listening study. Music Sci. 3, 205920432093570. (doi:10.1177/205920432093570)

[109] Rassouli M . 2026 Expressing Emotions Through Music – Supplementary Material. Retrieved from https://osf.io/69qwv.

[110] Putkinen V , Rassouli M, Nummenmaa L. 2026 Supplementary material from: Expressing emotions through music. Figshare. (doi:10.6084/m9.figshare.c.8646405) PMC13601867 42779434

