# Supplementary material for: Expressing emotions through music
Source: Philos Trans R Soc Lond B Biol Sci. 2026 Sep 24;381(1959):20250131. doi: 10.1098/rstb.2025.0131 (PMC13601867; doi:10.1098/rstb.2025.0131)
Supplement: Supplementary file 1 [file rstb.2025.0131.zip › rstb.2025.0131/rstb20250131_si_001.docx]

**SUPPLEMENTARY MATERIAL FOR**

Expressing Emotions Through Music

Vesa Putkinen^1,2^ Maya Rassouli^1,2^ and Lauri Nummenmaa^1,2,3^

^1^Turku PET Centre, University of Turku, Finland

^2^Turku University Hospital, University of Turku, Finland

^3^Department of Psychology, University of Turku, Finland

## Methods

**Literature Search**

A systematic search was conducted in PubMed and Web of Science for articles published between January, 1st 2000 and August, 6th 2025. The search strategy for publications reporting neurofunctional correlates of music making was developed using the following search strings in PubMed:

1. *(“music” [All Fields] AND (“generation” [All Fields] OR “playing” [All Fields] OR “performance” [All Fields] OR “creation” [All Fields] OR “production” [All Fields] OR “compose” [All Fields])) AND “fMRI” [All Fields]*, and
2. *(“music” [All Fields] AND (“generation” [All Fields] OR “playing” [All Fields] OR “performance” [All Fields] OR “creation” [All Fields] OR “production” [All Fields] OR “compose” [All Fields])) AND “PET” [All Fields]*.

In Web of Science, similar search strings were generated as follows

1. *“music” AND “fmri” AND “generation”,*
2. *“music” AND “fMRI” AND “playing”,*
3. *“music” AND “fMRI” AND “performance”,*
4. *“music” AND “fMRI” AND “creation”,*
5. *“music” AND “fMRI” AND “production”,*
6. *“music” AND “fMRI” AND “compose”,*
7. *“music” AND “PET” AND “generation”,*
8. *“music” AND “PET” AND “playing”,*
9. *“music” AND “PET” AND “performance”,*
10. *“music” AND “PET” AND “creation”,*
11. *“music” AND “PET” AND “production”,* and
12. *“music” AND “PET” AND “compose”*.

**Screening**

First, articles were screened by title and abstract, as well as a quick look at full-text. At this stage, the **inclusion criteria** were as follows:

1. Full text of the article was available (i.e. conference abstracts were excluded).
2. The article was published in a peer-reviewed journal.
3. The article presented original empirical data rather than a review or a meta-analysis.
4. The study specifically investigated the neural effects of actively producing music (audiomotor task) during the act of playing using (f)MRI or PET, regardless of musical instrument/activity and participants’ characteristics (trained musicians or non-musicians, age, gender).

Next, full texts were evaluated and articles were excluded if

1. Coordinate-based results (MNI or Talairach) were not reported or available
2. The tasks were not related to actual musical performance, i,.e. studies on observation of musical performance, imagined performance, musical performance without auditory feedback, motor sequences, and tapping to the beat were excluded.

**Data extraction**

The following information were extracted from each publication: title, authors, year of publication, number of participants, age, sex, characteristics of participants (musicians or non-musicians), description of the tasks, musical instrument played, imaging modality (PET or fMRI) (**Table S1 and Table S2**). The reported coordinates for each contrast were manually extracted from each publication and reported in a table. Alongside each extracted coordinate, the type of contrast, the stereotactic space, the sample size, the instrument played/musical activity performed, the imaging modality, and whether the subjects were musicians, non-musicians or both were reported. The data were double-checked by the same investigator (MR).

Only results based on whole-brain analysis were included, and results based on Region-Of-Interest (ROI) analysis or connectivity analysis were not included. Additionally, discussions between two investigators (MR and VP) established further criteria as to what to include or exclude as well as to how to report the studies. Main effects of musical tasks (musical task > rest, or musical task > non-musical task) as well as comparison between two musical tasks were reported in the table (musical task 1 > musical task 2, and musical musical task 2 > musical task 1). A coordinate was regarded as a deactivation foci and marked as such in the table when the contrast studied compared a non-musical task or a baseline condition with a musical task (non-musical task > musical task, and rest > musical task). However, deactivation foci were later excluded from activation likelihood estimation (ALE) analysis.

Coordinates describing the conjunction of a musical task and a non-musical task together were excluded. Coordinates reported for different participant groups but for the same contrasts within an article were regarded as separate studies. Contrasts regarded as redundant (i.e. same data were reused across related contrasts) were discarded. Finally, dependencies were avoided by regarding separate articles using the same exact participants as one study, and in case an article reported different contrasts for the separate groups than the reported ones for both groups together, deciding which contrasts were more meaningful in the context of the meta-analysis and excluding the other.

A total of 1659 articles (including duplicates) were identified through database searching and initially screened by title and abstract. Then, 106 articles were assessed for eligibility in the in depth full-text screening stage. From these, **40** articles fulfilled criteria for eligibility and altogether **848** participants and **1727** activation foci were included in the meta-analysis. Neuroimaging method mainly comprised fMRI (97.5%). Only a single study used PET imaging (2.5%). Musical instrument and musical activity varied across studies including keyboard (47.5%), vocal activity (27.5%), rhythmic activity (17.5%), conducting (2.5%), cube (2.5%), and finger force sensor (2.5%) (**Table S1**). Musicians were reported in 62,26% of the included studies and non-musicians in 37,74% (**Table S2**).

**Activation Likelihood Estimation (ALE)**

To identify the neural correlates of music production in individuals, **coordinate-based meta-analysis** (CBMA) of the selected studies was conducted. Specifically, the activation likelihood estimation (ALE) method (Eickhoff et al., 2012) implemented in NiMARE v0.0.7 (Salo et al., 2021, 2023) was used to evaluate the convergence of peak coordinates. If necessary, coordinates were converted from Talairach to MNI space using the Lancaster transform (tal2mni() function) incorporated in NiMARE. The ALE method convolves each peak from each study with a 3D Gaussian distribution determined by the study’s sample size, and combines the resulting activation maps across studies into an ALE map, which is then compared with an empirical null distribution (Salo et al., 2023). Statistical significance was estimated using Monte Carlo method with 10 000 iterations and the results were thresholded using cluster-level inference at p < 0.01 (FWE). Results were visualized with the software MRIcroGL (<https://www.nitrc.org/projects/mricrogl/>).

**Functional Coactivation Analysis**

To assess functional coactivation, ROI-wise correlations between our meta-analytic neural map of music production and the neural networks associated with emotions, music, and music-induced emotions were calculated. The terms “emotions” and “music” were searched in NeuroSynth database (Yarkoni et al., 2011), and the thresholded resultant uniformity test maps (p < 0.01, FDR corrected) were retrieved (respectively, on October 9th 2025 and on October 15th 2025). Additionally, the ALE meta-analytic map generated for music-induced emotions by Nummenmaa and colleagues (2021) (p < 0.05, FWE corrected) was also used for comparisons. Visualization of the maps were made with the software MRIcroGL (<https://www.nitrc.org/projects/mricrogl/>) (**Figure 1**). Each retrieved map was Pearson correlated with our own generated -log10(p) map within 14 selected ROIs. The ROIs included caudate, putamen, nucleus accumbens, amygdala, insula, thalamus, anterior cingulate cortices (ACC), orbitofrontal cortex (OFC), supplementary motor area (SMA), postcentral gyrus (POSCG), precentral gyrus (PRECG), superior temporal gyrus (STG), middle temporal gyrus (MTG), and Heschl’s gyrus (HG).

## Table S1 Characteristics of Studies Included in the Meta-Analysis

| **study_ids** | **Title** | **Authors** | **Year** | **Method** | **Musical Instrument** | **Tasks** |
| --- | --- | --- | --- | --- | --- | --- |
| **study_005** | The brain basis of piano performance | Lawrence M. Parsons, Justine Sergent, Donald A. Hodges, Peter T. Fox | 2005 | PET | keyboard | playing scales and Bach on keyboard |
| **study_006** | Dissociation between melodic and rhythmic processing during piano performance from musical scores | Sara L. Bengtsson, Fredrik Ullén | 2006 | fMRI | keyboard | playing 1) combined (melodic and rhythmic structures), 2) melodic structure with an isochronous rhythm, 3) rhythmic structure, and 4) single pitch sequence with an isochronous rhythm on keyboard |
| **study_009** | Cerebral networks for spontaneous and synchronized singing and speaking | Yoko Saito, Kenjo Ishii, Kazuo Yagi, Itaru F Tatsumi, Hidehiro Mizusawa | 2006 | fMRI | singing | 1) overtly singing ‘Umi’ (The Sea) – alone (Sg_solo); 2) overtly singing Umi in synchronization with the auditory presentation of the song, including the melody and lyrics (Sg_syn); 3) listening to the singing undertaken in the previous task (L_Sg); 4) overtly reciting alone the lyrics of Umi without the melody (Sp_solo); 5) overtly reciting Umi along with an auditory presentation of the lyrics (Sp_syn); 6) listening to the reciting undertaken in the previous task (L_Sp); and 7) rest condition. |
| **study_006** | Cortical Regions involved in the generation of musical structures during improvisation in pianists | Sara L. Bengtsson, Mihaly Csikszentmihaly, Fredrik Ullén | 2007 | fMRI | keyboard | 1) Improvise on the basis of a visually displayed melody. 2) Reproduce its previous improvisation from memory on the keyboard |
| **study_011** | overt and imagined singing of an Italian aria | B Kleber, N Birbaumer, R Veit, T Trevorrow, M Lotze | 2007 | fMRI | singing | Singing and imagining singing phrases taken from the first line of an Italian aria |
| **study_013** | Generation of novel motor sequences: the neural correlates of musical improvisation | Aaron L. Berkowitz, Daniel Ansari | 2008 | fMRI | keyboard | Playing 1) Patterns (ascending, descending or 5-sequential presses of any key) with metronome 2) Melodic improvisation with metronome 3) Patterns with chosen rhythm 4) Melodic improvisation with chosen rhythm on keyboard |
| **study_014** | Neural substrates of spontaneous musical performance: an fMRI study of jazz improvisation | Charles J. Limb, Allen R. Braun | 2008 | fMRI | keyboard | Playing 1) scales 2) improvised melody with scale restriction 3) pre-learned jazz composition 4) improvised jazz-style composition on keyboard |
| **study_016, study_016a** | Experience-dependent neural substrates involved in vocal pitch regulation during singing | Jean Mary Zarate, Robert J Zatorre | 2008 | fMRI | singing | Non-musicians or singers singing a single tone or listening to it |
| **study_013** | Expertise-related deactivation of the right temporoparietal junction during musical improvisation | Aaron L. Berkowitz, Daniel Ansari | 2010 | fMRI | keyboard | Playing 1) Patterns (ascending, descending or 5-sequential presses of any key) with metronome 2) Melodic improvisation with metronome 3) Patterns with chosen rhythm 4) Melodic improvisation with chosen rhythm on keyboard |
| **study_018** | Novel approach for understanding the neural mechanisms of auditory-motor control:pitch regulation by finger force | Ryosuke O. Tachibana, Masuzo Yanagida, Hiroshi Riquimaroux | 2010 | fMRI | finger force sensor | (AM) Match the pitch of feedback tone from finger force sensor to target tone, (A) Listen to tone bursts, (M) Press the sensor with finger force to match the instruction tone but without audiofeedback |
| **study_019** | Vocal accuracy and neural plasticity following micromelody-discrimination training | Jean Mary Zaratem Karine Delhommeau, Sean Wood, Robert J. Zatorre | 2010 | fMRI | singing | Singing: singing single-note and melodies, Voice perception task : listening to target tone and melody |
| **study_020** | Learning to play a melody: an fMRI study examining the formation of auditory-motor associations | Joyce L. Chen, Charlotte Rae, Kate E. Watkins | 2012 | fMRI | keyboard | Listening to and playing a melody on keyboard 5 times in a row (learning melody), listening to and playing a random melody on keyboard different each time (random learning) |
| **study_022** | Goal-independent mechanisms for free response generation: creative and pseudo-random performance share neural substrates | Örjan de Manzano, Fredrik Ullén | 2012 | fMRI | keyboard | Improvise : do musical improvisations, Random : press the keys in a random fashion, Notes : try and reproduce the musical scores as accurately as possible on the keyboard |
| **study_023** | Neural correlates of lyrical improvisation: an fMRI study of freestyle Rap | Siyuan Liu, Ho Ming Chow, Yisheng Xu, Michael G. Erkkinen, Katherine E. Swett, Michael W. Eagle, Daniel A. Rizik-Baer, Allen R. Braun | 2012 | fMRI | freestyle rap | Improvise : improvise rap lyrics, Conventional : rap the memorized lyrics on the same instrumental track |
| **study_024** | Repetition suppression in auditory-motor regions to pitch and temporal structure in music | Rachel M. Brown, Joyce L. Chen, Avrum Hollinger, Virginia B. Penhune, Caroline Palmer, Robert J Zatorre | 2013 | fMRI | keyboard | Playing back a melody they listened to on a keyboard 1) No repeat: the pitch and the timing sequence changed 2) All repeat: the pitch and the timing remained constant 3) Pitch repeat: only the timing sequence changed 4) Timing repeat: only the pitch changed |
| **study_26b** | Experience-dependent modulation of feedback integration during singing: role of the right anterior insula | Boris Kleber, Anthony G. Zeitouni, Anders Friberg, Robert J. Zatorre | 2013 | fMRI | singing | Singing musical target intervals under normal conditions and after anesthesia |
| **study_027, study_027a** | Neural correlates of musical creativity: differences between high and low creative subjects | Mirta F. Villarreal, Daniel Cerquetti, Silvina Caruso, Violeta Schwarcz López Aranguren, Eliana Roldán Gerschcovich, Ana Lucía Frega, Ramón C. Leiguarda | 2013 | fMRI | response box button | Create a new rhythm on a response box based on a rhythm they just listened to; Repeat the rhythm they just heard |
| **study_029** | Neural substrates of interactive musical improvisation: an fMRI study of 'Trading Fours' in Jazz | Gabriel F. Donnay, Summer K. Rankin, Monica Lopez-Gonzalez, Patpong Jiradejvong, Charles J.Limb | 2014 | fMRI | keyboard | Scale - Control: subject A (in the scanner) and B alternated playing a D Dorian scale in quarter notes on keyboard; Scale - Improv: subject A and subject B took turns improvising 4 measure phrases. Jazz - Control: subject A and subject B alternated playing 4 measure segments of a novel jazz composition memorized previously; Jazz - Improv : subject A and subject B traded 4 (took turns improvising 4 measure phrases). |
| **study_030** | Brain responses to altered auditory feedback during musical keyboard production: an fMRI study | Peter Q. Pfordresher, James T. Mantell, Steven Brown, Robert ZIvadinov, Jennifer L. Cox | 2014 | fMRI | keyboard | normal : perform melodies with normal feedback on keyboard, silent : perform with no feedback, listen : listen to the melody without performing, asynchrony : delayed to be 33% of the running average produced inter-response intervals (IRIs), shifted : each key press would trigger the pitch corresponding to the previous key press, random : each key press would trigger a random pitch |
| **study_031** | Connecting to create: expertise in musical improvisation is associated with increased functional connectivity between premotor and prefrontal areas | Ana Luísa Pinho, Örjan de Manzano, Peter Fransson, Helene Eriksson, Fredrik Ullén | 2014 | fMRI | keyboard | improvise on keyboard with different constraints |
| **study_033** | Neural activity related to discrimination and vocal production of consonant and dissonant musical intervals | Nadia González-Garcia, Martha A Gonz´lez, Pablo L Rendón | 2016 | fMRI | singing | singing 2s target interval or passively listening to a musical interval |
| **study_034** | Emotional Intent Modulates the Neural substrates of creativity: an fMRI study of emotionally targeted Improvisation in Jazz musicians | Malinda J. McPherson, Frederick S. Barrett, Monica Lopez-Gonzalez, Patpong Jiradejvong, Charles J. Limb | 2016 | fMRI | keyboard | view : simply look at the image (negative, ambiguous, positive face), chromatic scale : play an ascending and descending chromatic scale over the entire range of the keyboard at the same tempo regardless of the picture, improvise : improvise a composition that they felt best represented the emotion expressed by the images |
| **study_038, study_038a** | Efficacy of auditory versus motor learning for skilled and novice performers | Rachel M Brown, Virginia B Penhune | 2018 | fMRI | keyboard | During learning trial : Cue : viewing the visual cues corresponding to the melody without performing or listening to the melody, auditory : viewing the visual cues while listening to the melody, motor : viewing the visual cues while performing the melody without listening to the melody, auditory-motor : viewing the cues while performing the melody and hearing auditory feedback. During recall trial : performing the melody from memory while hearing auditory feedback with no visual cues present on keyboard |
| **study_039** | The language of music: common neural codes for structured sequences in music and natural language | Jeffrey N Chiang, Matthew H Rosenberg, Carolyn A Bufford, Daniel Stephpens, Antonio LYSY, Martin M Monti | 2018 | fMRI | singing | MUSIC: structure : sing the root position or the IInd inversion of a musical note, repeat : repeat the musical note. LANGUAGE : structure : form an active or passive sentence with word, repeat : repeat the word |
| **study_041, study_041a** | Different role of the supplementary motor area and the insula between musicians and non-musicians in a controlled musical creativity task | Marcella Pereira Barbosa de Aquino, Juan Verdejo-Román, Miguel Pérez-García, Purificación Pérez-García | 2019 | fMRI | touchpad | Improvise : play a new rhythm employing any kind of modification of the presented rhythm score (that they had just heard). Repeat : reproduce the rhythm that they had just heard on an evoke response pad system. Rest |
| **study_042** | Higher Node Activity with Less Functional Connectivity During Musical Improvisation | Kiran Dhakal, Martin Norgaard, Bhim M Adhikari, Kristy S Yun, Mukesh Dhamala | 2019 | fMRI | singing | VP vocalize prelearned : vocalize familiar melody; IP imagine prelearned : imagine familiar melody; VI vocalize improvised :vocalize a spontaneously improvised melody over the blues chord progression; II imagine improvised : imagine a spontaneously improvised melody over the blues chord progression |
| **study_043** | How task interactivity shapes action observation | L.M. Sacheli, C. Verga, E. Arcangeli, G. Banfi, M. Tettamanti, E. Paulesu | 2019 | fMRI | cube | TASK : Joint action : playing a four-note melody together with their partner, in alternating turns of playing one note each. Non-Interactive : play one of four pairs of notes independent of the notes that their partner is playing on a cube. TRIAL TYPE : Trial 1 : first half of the musical sequence. Trial 2 : second half. CONGRUENCE : congruent : imitative (pointing action in response to partner's pointing action). incongruent : non-imitative (grasping in response to partner's pointing action) |
| **study_044** | Classical creativity: a functional magnetic resonance imaging (fMRI) investigation of pianist and improviser Gabriela Montero | Karen Chan Barrett, Frederick Streeter Barrett, Patpong Jiradejvong, Summer K Rankin, Andrew T Landau, Charles J Limb | 2020 | fMRI | keyboard | improvise : improvise upon composition used as control (chromatic scale or a pre-learned memorized composition) on keyboard |
| **study_045** | Mental Effort When Playing, Listening, and Imagining Music in One Pianist's Eyes and Brain | Tor Endestad, Rolf Inge Godoy, Markus Handal Sneve, Thomas Hagen, Agata Bochynska, Bruno Laeng | 2020 | fMRI | keyboard | listening (listening to a music clip while viewing the music sheet), imagining the sounds ( imagine hearing the melody in her head), imagining playing (imagine playing the same piece without moving), and actually playing (play the music from the presented score sheet using the keyboard). 3 piano pieces of different technical and interpretive difficulty: an easy piece, a middle-level one, and an advanced one. |
| **study_049** | Cortico-cerebellar audio-motor regions coordinate self and other in musical joint action | Natalie Kohler, Giacomo Novembre, Katarzyna Gugnowska, Peter E Keller, Arno Villringer, Daniela Sammler | 2023 | fMRI | keyboard | pianist A (melody) plays piano in the MR scanner in duet with pianist B (bassline) outside the scanner. familiar : both parts were familiar to the pianist. unfamiliar : their partner's respective part was unfamiliar. congruent : pianists received the same cue for tempo. incongruent : pianists received opposite cues for tempo. |
| **study_050** | Differential effects of ageing on the neural processing of speech and singing production | Nella Moisseinen, Teppo Särkämo, Jaak Kauramäki, Boris Kleber, Aleksi J Sihvonen, Noelia Martínez-Molina | 2023 | fMRI | singing | speech : proverbs, singing : sing phrases, repetition : repeat familiar new proverbs and song phrases, completion : complete familiar new proverbs and song phrases from memory, improvisation : improvise new proverbs and song phrases |
| **study_051** | Speaking in gestures: left dorsal and ventral frontotemporal brain systems underlie communication in conducting | Mariacristina Musso, Eckart Altenmüller, Marco Reisert, Jonas Hosp, Ralp Schwarzwald, Bettina Blank, Julian Horn, Volkmar Glauche, Christoph Kaller, Cornelius Weiller, Martin Schumacher | 2023 | fMRI | conducting | C-task : conducting the musical tracks by moving the baton to set the musical tempo; L-task : listening to the same musical tracks, Cf-task : conducting the musical tracks without changing the tempo; M-task : performing the beat patterns of a metronome beating the same tempo as the musical track, D-task: performing beat patterns after identifying the temporal patterns of a drum playing sequence with complex rhythmic structures |
| **study_052** | Unlocking the musical brain: A proof-of-concept study on playing the piano in MRI scanner with naturalistic stimuli | Alicja M Olszewska, Dawid Dro´zdziel, Maciej Gaca, Agnieszka Kulesza, Wojciech Obrębski, Jakub Kowalewski, Agnieszka Widlarz, Artur Marchewka, Aleksandra M Herman | 2023 | MRI | keyboard | listen : listen to a fragment of a prelearned melody; playback : play the fragment just previously heard either with right hand only or with two hands on keyboard. listen : listening to heptatonic scale played forwards and backwards; playback : repeat the scale just heard and in half of the trials the auditory feedback on one of the heard keys' pitch was modulated by a halftone or a whole tone. |
| **study_053** | The rhythmic mind: brain functions of percussionists in improvisation | Yin-Chun Liao, Ching-Ju Yang, Hsin-Yen Yu, Chiu-Jung Huang, Tzu-Yi Hong, Wei-Chi Li, Li-Fen Cen, Jen-Chuen Hsieh | 2024 | fMRI | touchpad | SR : sight-reading, tap the rhythm of just viewed/heard percussion score accompanied by the sound beat on a touchpad using their index fingers; SIMP : structured improvisation based on rhythmic patterns of 6 examples; FIMP : free improvisation on touchpad |
| **study_054** | Children engage neural reward structures for creative musical improvisation | Karen Chan Barrett, Patpong Jiradejvong, Lauren Jacobs, Charles J Limb | 2025 | fMRI | keyboard | scale control : play pentatonic scale up and down to the provided beat on keyboard; impro : play same notes to the beat but in whatever order desired |
| **study_055** | The right sound at the right time: Cerebellar and ventral striatal involvement in imitating pitch and timing | M. Belyk, S.A. Kotz | 2025 | fMRI | singing, whistling | listen : listen to 5-note sound sequence, sing/whistle note sequences, half of the stimuli contained melodies that varied in pitch but held note durations constant, half contained note sequences in which the note durations varied but pitch was held constant. |
| **study_057, study_057a** | Moving on Time: Brain Network for Auditory-Motor Synchronization is modulated by rhythm complexity and musical training | Joyce L. Chen, Virginia B. Penhune, Robert J. Zatorre | 2008 | fMRI | computer mouse key | listen and imitate auditory rhythms by tapping in synchrony on a computer mouse |
| **study_058** | Rhythm information represented in the fronto-parieto-cerebellar motor system | Naho Konoike, Yuka Kotozaki, Shigehiro Miyachi, Carlos Makoto Miyauchi, Yukihito Yomogida, Yoritaka Akimoto, Koji Kuraoka, Motoaki Sugiura, Ryuta Kawashima, Katsuki Nakamura | 2012 | fMRI | finger tapping | rhythm : listen/visualize a rhythm pattern and reproduce it by tapping it, number : listen/visualize sequence and tap the number of stimuli in the sequence (regardless of tempo) |
| **study_060** | Inner sense of rhythm: percussionist brain activity during rhythmic encoding and synchronization | Yin-Chun Liao, Ching-Ju Yang, Hsin-Yen Yu, Chiu-Jung Huang, Tzu-Yi Hong, Wei-Chi Li, Li-Fen Cen, Jen-Chuen Hsieh | 2024 | fMRI | touchpad | SynC : synchronize their tapping to a rhythm of 100 beats per minute |
| **study_061** | When direction matters: neural correlates of interlimb coordination of rhythm and beat | Signe H Marup, Boris A Kleber, Cecilie Moller, Peter Vuust | 2024 | fMRI | tapping | perform a repeated rhythmic pattern using one effector and its underlying beat using another effector (voice, right hand, left hand, right foot). Each effector combination is performed either against (aH) the bodily hierarchy, with bodily hierarchy (wH) or as control condition with the beat in both effectors (isochronous baseline) |

## Table S2 Characteristics of Participants in the Studies Included in the Meta-Analysis

| **study_ids** | **Musicians** | | | | | **Non-Musicians** | | | | |
| --- | --- | --- | --- | --- | --- | --- | --- | --- | --- | --- |
|  | **n** | **Females** | **Males** | **Age mean ± SD** | **Age range** | **n** | **Females** | **Males** | **Age mean ± SD** | **Age range** |
| **study_005** | 8 | 5 | 3 | - | 27-54 | - | - | - | - | - |
| **study_006** | 11 | 0 | 11 | 32 ± 6 | 23-41 | - | - | - | - | - |
| **study_009** | - | - | - | - | - | 20 | 10 | 10 | - | 19-24 |
| **study_011** | 16 | 11 | 5 | 31.06 ± 8.27 | 20-44 | - | - | - | - | - |
| **study_013** | 13 (1 excluded) | 8 | 5 | 21.9 | - | - | - | - | - | - |
| **study_014** | 6 | 0 | 6 | 34.2 ± 10.4 | 21-50 | - | - | - | - | - |
| **study_016** | - | - | - | - | - | 12 | 6 | 6 | - | - |
| **study_016a** | 12 | 6 | 6 | - | - | - | - | - | - | - |
| **study_018** | 12 | 5 | 7 | - | 21-27 | - | - | - | - | - |
| **study_019** | - | - | - | - | - | 20 | 12 | 8 | 22 ± 4.4 | - |
| **study_020** | - | - | - | - | - | 16 | 8 | 8 | 27.13 | 20-34 |
| **study_022** | 18 | 1 | 17 | 39 ± 12 | - | - | - | - | - | - |
| **study_023** | 12 | 0 | 12 | 30.3 | 23-36 | - | - | - | - | - |
| **study_024** | 14 | 4 | 10 | 21.88 | 18-29 | - | - | - | - | - |
| **study_26b** | 17 (3 excluded) | 12 | 5 | 33 | - | 12 | 6 | 6 | 26 | - |
| **study_027, study_027a** | 24 | 15 | 9 | 21.9 ± 1.6 | - | - | - | - | - | - |
| **study_029** | 11 | 0 | 11 | 38.8 ± 11 | 25-6 | - | - | - | - | - |
| **study_030** | 20 (1 excluded) | 16 | 4 | 24 | 19-35 | - | - | - | - | - |
| **study_031** | 39 | 15 | 24 | 32.4 ± 11 | 19-67 | - | - | - | - | - |
| **study_033** | 11 | 11 | 0 |  | 15-21 | - | - | - | - | - |
| **study_034** | 12 | 1 | 11 | 39.9 ± 15.8 | - | - | - | - | - | - |
| **study_038** | 16 | 8 | 8 | 23.1 | - | - | - | - | - | - |
| **study_038a** | - | - | - | - | - | 16 | 11 | 5 | 24.1 | - |
| **study_039** | 20 | 8 | 12 | - | - | - | - | - | - | - |
| **study_041** | 19 | 10 | 9 | 20.26 ± 2.05 | - | - | - | - | - | - |
| **study_041a** | - | - | - | - |  | 21 | 16 | 5 | 20.19 ± 2.36 | - |
| **study_042** | 24 | 0 | 24 | 31.9 ± 13.6 | - | - | - | - | - | - |
| **study_043** | - | - | - | - | - | 24 | 11 | 13 | 23.75 ± 2.02 | 19-27 |
| **study_044** | 1 | 1 | 0 | 44 | - | - | - | - | - | - |
| **study_045** | 1 | 1 | 0 | 41 | - | - | - | - | - | - |
| **study_049** | 40 (1 excluded) | 20 | 20 | 25.25 ± 5.30 | 18-39 | - | - | - | - | - |
| **study_050** | - | - | - | - | - | 100 | 55 | 45 | 49.2 ± 17.5 | 21-88 |
| **study_051** | 28 | 3 | 25 | - | - | - | - | - | - | - |
| **study_052** | 20 | 20 | 0 | 21.9 ± 2.1 | 19-26 | - | - | - | - | - |
| **study_053** | 25 | 15 | 10 | 23.92 ± 2.84 | - | - | - | - | - | - |
| **study_054** | - | - | - | - | - | 12 | 4 | 8 | - | 9-11 |
| **study_055** | - | - | - | - | - | 13 | 9 | 4 | 26.4 ± 5.2 | - |
| **study_057** | - | - | - | - | - | 12 | 6 | 6 | 23.83 | 20-32 |
| **study_057a** | 12 | 6 | 6 | 23.17 | 19-28 | - | - | - | - | - |
| **study_058** | - | - | - | - | - | 17 (1 excluded) | 7 | 10 | 21.9 | 18-28 |
| **study_060** | 26 | 15 | 11 | 24.1 ± 3 | - | 26 | 15 | 11 | 23.7 ± 2.8 | - |
| **study_061** | 46 | 15 | 31 | 27.28 ± 4.8 | - | - | - | - | - | - |

## References

Eickhoff, S. B., Bzdok, D., Laird, A. R., Kurth, F., & Fox, P. T. (2012). Activation Likelihood Estimation meta-analysis revisited. Neuroimage, 59(3), 2349–2361. <https://doi.org/10.1016/j.neuroimage.2011.09.017>

Nummenmaa, L., Putkinen, V., Sams, M. (2021). Social pleasures of music. Current Opinion in Behavioral Sciences, 39, 196-202. <https://doi.org/10.1016/j.cobeha.2021.03.026>.

Salo, T., Yarkoni, T., Nichols, T. E., Poline, J.-B., Bilgel, M., Bottenhorn, K. L., Eickhoff, S. B., Jarecka, D., Kent, J. D., Kimbler, A., Nielson, D. M., Oudyk, K. M., Peraza, J. A., Pérez, A., Reeders, P. C., Yanes, J. A., & Laird, A. R. (2023). NiMARE: Neuroimaging Meta-Analysis Research Environment. Aperture Neuro, 3, 1–32. <https://doi.org/10.52294/001C.87681>

Salo, T., Yarkoni, T., Nichols, T. E., Poline, J.-B., Kent, J. D., Gorgolewski, K. J., Glerean, E., Bottenhorn, K. L., Bilgel, M., Wright, J., Reeders, P., Kimbler, A., Nielson, D. N., Yanes, J. A., Pérez, A., Oudyk, K. M., Jarecka, D., & Laird, A. R. (2021). neurostuff/NiMARE: 0.0.7.

<https://doi.org/10.5281/ZENODO.4562954>

Yarkoni, T., Poldrack, R. A., Nichols, T. E., Van Essen, D. C., & Wager, T. D. (2011). Large-scale automated synthesis of human functional neuroimaging data. Nature Methods 2011 8:8, 8(8), 665–670. <https://doi.org/10.1038/nmeth.1635>
